# Quantitative diagnosis of HER2 protein expressing breast cancer by single‐particle quantum dot imaging

**DOI:** 10.1002/cam4.898

**Published:** 2016-09-26

**Authors:** Minoru Miyashita, Kohsuke Gonda, Hiroshi Tada, Mika Watanabe, Narufumi Kitamura, Takashi Kamei, Hironobu Sasano, Takanori Ishida, Noriaki Ohuchi

**Affiliations:** ^1^ Department of Surgical OncologyGraduate School of MedicineTohoku UniversitySeiryo‐machi, Aoba‐kuSendai980‐8574Japan; ^2^ Department of Nano‐Medical ScienceGraduate School of MedicineTohoku UniversitySeiryo‐machi, Aoba‐kuSendai980‐8575Japan; ^3^ Department of Medical PhysicsGraduate School of MedicineTohoku UniversitySeiryo‐machi, Aoba‐kuSendai980‐8575Japan; ^4^ Department of PathologyTohoku University HospitalSeiryo‐machi, Aoba‐kuSendai980‐8574Japan; ^5^ Department of Advanced Surgical Science and TechnologyGraduate School of MedicineTohoku UniversitySeiryo‐machi, Aoba‐kuSendai980‐8574Japan

**Keywords:** Breast cancer, HER2, quantum dot, single‐particle imaging, trastuzumab

## Abstract

Overexpression of HER2 is one of the major causes of breast cancer, and therefore precise diagnosis of its protein expression level is important. However, current methods estimating the HER2‐expression level are insufficient due to problem with the lack of quantification. This might result in a gap between diagnostics and therapeutics targeting HER2. Therefore, a new effective diagnostic method is needed. We developed a new immunohistochemical (IHC) technique with quantum dots (QD)‐conjugated trastuzumab using single‐particle imaging to quantitatively measure the HER2 expression level. Tissues from 37 breast cancer patients with available detailed clinical information were tested by IHC with QDs (IHC‐QD) and the correlation with IHC with 3,3′‐diaminobenzidine (DAB), fluorescence in situ hybridization (FISH), and IHC‐QD was examined. The number of QD‐conjugated trastuzumab particles binding specifically to a cancer cell was precisely calculated as the IHC‐QD score. The IHC‐QD score in 37 cases was correlated proportionally with the score of HER2 gene copy number as assessed by FISH (*R* = 0.83). When HER2 positivity was judged to be positive, the IHC‐QD score with our cut‐off level was exactly concordant with the FISH score with a cut‐off value of 2.0. Furthermore, IHC‐QDs score and time to progression (TTP) of trastuzumab therapy were well correlated in HER2‐positive cases (*R* = 0.69). Conversely, the correlation between FISH score and TTP was not observed. We developed a precisely quantitative IHC method using trastuzumab‐conjugated QDs and single‐particle imaging analysis and propose the possibility of using IHC‐QDs score as a predictive factor for trastuzumab therapy.

## Introduction

A total of 15–20% of patients with breast cancer have overexpression of human epidermal growth factor receptor 2 (HER2)/neu in their tumors. HER2‐positive status is correlated with aggressive and poorly differentiated tumors and results in a worse prognosis 1, 2. Trastuzumab is a humanized monoclonal antibody against the HER2 protein and contributes to improvements of the clinical outcome of these patients 3, 4, 5. Recently, in addition to trastuzumab, the new anticancer drugs trastuzumab‐emtansine and pertuzumab were developed against HER2 6, 7, 8. Thus, diagnostic accuracy in detecting patients who are HER2‐positive is clinically significant for treatment with trastuzumab. In most HER2‐positive patients, HER2 gene amplification on chromosome 17 causes overexpression of the protein 6. Until now, HER2 gene amplification and its protein overexpression have been measured by immunohistochemistry (IHC) and fluorescence in situ hybridization (FISH).

**Figure 1:**
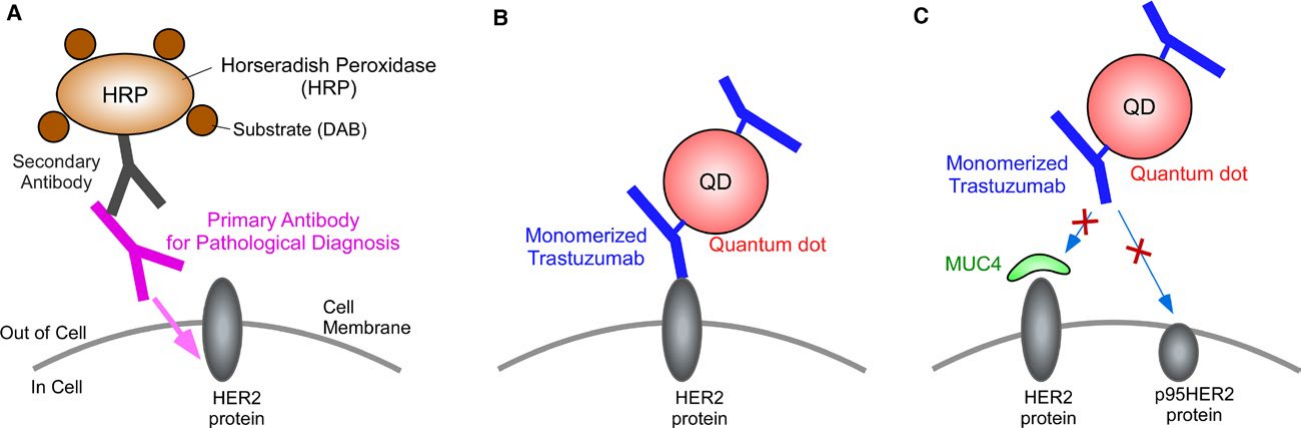
Schematic drawing of IHC with DAB (IHC‐DAB) and IHC with quantum dots (IHC‐QDs). In conventional IHC‐DAB, HER2 proteins are immunostained with primary antibody and secondary antibody conjugated with HRP (A). The primary antibodies used for pathological diagnosis of trastuzumab therapy recognize intracellular domain of HER2 protein (A). On the other hand, trastuzumab recognizes the extracellular domain of HER2 (B). Therefore, epitope of trastuzumab differs from that of diagnostic antibodies. In IHC‐QDs, trastuzumab were monomerized and then conjugated with QDs (mean value, 2.5 of monomer trastuzumab fragments per single QD) (B). It have been reported that various truncated forms of HER2 lack the extracellular domain, like p95HER2 (C). Trastuzumab‐conjugated QDs cannot bind to the truncated forms of HER2 (C). In addition, overexpression of MUC4 is known to seal the surface of the HER2 protein. These effects on the extracellular domain of HER2 prevent the interaction of trastuzumab with HER2 but not the interaction of a diagnostic antibody and HER2 (C). The difference of epitope between antibodies leads to the gap between diagnostics and therapeutic efficacy. DAB, 3,3′‐diaminobenzidine; HER2, human epidermal growth factor receptor 2; IHC, immunohistochemical; HRP, horseradish peroxidase.

IHC with 3,3′‐diaminobenzidine (DAB) (IHC‐DAB), the most conventional IHC protocol 9, 10, 11, has two disadvantages. First, IHC‐DAB is not quantitative, whereas FISH can quantitatively estimate the gene copy number. In IHC‐DAB, the intensity of DAB staining depends on the enzymatic activity of horseradish peroxidase (HRP). Therefore, the staining intensity of DAB is significantly influenced by the reaction time, temperature, and HRP substrate concentrations (Fig. 1A). IHC‐DAB against HER2 is classified into only four categories (scores of 0, 1, 2, and 3); furthermore, these categories are not based on quantitative amounts of HER protein. According to the recommended practice guideline for HER2 testing, negative for HER2 is defined as IHC‐DAB scores of 0–1+, equivocal for HER2 is defined as IHC‐DAB scores of 2+, and positive for HER2 is defined as IHC‐DAB scores of 3+. In cases of score 2+, FISH is required to judge whether HER2 positive or negative 12. Second, the epitopes of trastuzumab and most antibodies used for IHC‐DAB are different. Trastuzumab recognizes the extracellular domain of HER2, whereas the antibodies used for IHC‐DAB recognize its intracellular domain. Various truncated forms of HER2 that lack the extracellular domain have recently been reported. Other studies have shown that overexpression of MUC4 sealed the surface of the HER2 receptor 13, 14, 15. These effects on the extracellular domain of HER2 might inhibit the binding of trastuzumab to HER2 but not the binding of a diagnostic antibody and HER2 (Fig. 1A–C). Thus, to precisely estimate the affinity of trastuzumab to HER2, IHC using trastuzumab is necessary 16, 17, 18. In the current reports, about 70% of HER2‐positive patients show resistance to trastuzumab and experience disease progress during trastuzumab treatment 3. This response rate is not good enough for specific molecular‐targeted agents. The different epitopes among antibodies might lead to the gap between diagnostics and therapeutic efficacy.

Recent studies have focused on quantum dots (QDs) conjugated to anti‐HER2 antibodies 19, 20, 21, 22. Rakovich et al. showed that conjugating QDs to the single variable domain of anti‐HER2 antibodies can be successfully used for immunolabeling breast cancer cells 19. This can be a potential biomarker that is more sensitive than conventional immunohistochemistry processes. Rizvi et al. performed the bioconjugation of near‐infrared QDs to anti‐HER2 antibodies using an N‐ethyl‐N’‐(3‐dimethylaminopropyl)carbodiimide (EDC)/N‐hydroxysuccinimide (NHS) coupling method, and HER2 receptors were successfully localized in both fixed and live cancer cells 20. In previous studies, the prognostic value of immunofluorescent HER2 or Ki67 imaging was assessed using QD‐conjugated antibodies and human tissue samples 21, 22.

We also developed a new IHC with high quantitative sensitivity using autofluorescence‐subtracted images and single‐particle QD imaging 23, as illustrated in Figure 1. In this study, by application of this IHC, we developed an IHC method with trastuzumab conjugated with QDs, using tissue samples from patients with detailed clinical information. The score of IHC with QDs (IHC‐QDs) correlated proportionally with the FISH score and is a remarkable predictive factor for trastuzumab therapy in patients with breast cancer.

## Materials and Methods

### Patients and breast tissue specimens

We identified 798 patients with primary invasive breast cancer who underwent surgery at the Department of Surgery, Tohoku University Hospital (Sendai, Japan) between January 1998 and December 2008. The HER2 status for the 787 patients was obtained using conventional immunohistochemistry (IHC) using 3,3′‐diaminobenzidine (DAB) (IHC‐DAB). The distribution of HER2 status by IHC‐DAB scores of 0, 1, 2, and 3 was 52.2%, 26.8%, 7.8%, and 13.2%, respectively. We randomly selected 37 breast cancer specimens from 787 patients whose distribution by IHC‐DAB was 6, 6, 11, and 14 cases with scores of 0, 1, 2, and 3, respectively. Clinical information including response to trastuzumab treatment was retrieved through the breast cancer management database of the hospital. Response criteria is based on RECIST guideline, complete response (CR), partial response (PR), stable disease (SD), and progression disease (PD) 24. Therapeutic efficacy was evaluated by time to progression (TTP) defined as the time from the initiation of therapy to progression 25.

### Ethics statement

The Ethical Committee of the Graduate School of Medicine, Tohoku University, approved the protocol. All the patients signed an Ethical Committee consent form agreeing to serve as tissues donors for the experiments. The methods were performed in accordance with the approved guidelines.

### HER2 testing by IHC‐DAB and FISH

We confirmed the HER2 immunohistochemical expression of 37 cases using standard procedures on 3‐*μ*m‐thick sections of 10% formalin‐fixed and paraffin‐embedded breast tissue specimens and a kit for IHC‐DAB (HercepTest, Dako, Glostrup, Denmark). The results of IHC‐DAB were scored by two pathologists as follows: score 0 represents no staining or incomplete membrane staining in 10% or less of tumor cells; score 1 represents incomplete membrane staining in more than 10% of tumor cells; score 2 represents weak to moderate complete membrane staining in more than 10% of tumor cells or complete membrane staining in 10% or less of tumor cells; and score 3 represents intense circumferential membrane staining in more than 10% of tumor cells 12. All tumors were tested for gene amplification by FISH labeling the targeted DNA (the PathVysion HER2 DNA Probe Kit; Abbott, Chicago, IL). Slides were hybridized with probes to HER2/neu and CEP17, a marker of the centromere, using the PathVysion HER‐2 DNA Probe Kit (Abbott) according to the manufacturer's instructions. Sections were counterstained with 4,6‐diamidino‐2‐phenylindole and were visualized with a fluorescent microscope. The HER2 gene‐to‐CEP17 gene ratio was calculated according to the manufacturer's guidelines. A FISH score of 2.0 or above was defined as a HER2‐positive sample 12. A FISH score <2.0 and an average HER2 copy number of 6.0 or above was defined as a HER2‐positive tumor 12.

### QD‐conjugated trastuzumab

We prepared two kinds of quantum dot (QD)‐conjugated antibody complexes for IHC with QDs (IHC‐QDs). One was a QD‐conjugated trastuzumab (Chugai Pharmaceuticals Co. Ltd., Tokyo, Japan) 26, the other was a QD‐conjugated human IgG (Beckman Coulter, Inc., Fullerton, CA). These antibody were monomerized, mixed with QDs in a molar ratio of approximately 3: 1 (antibody: QDs), and then applied to a preparation of antibody‐conjugated QDs using a Qdot 705 Antibody Conjugation Kit (Life Technologies, Waltham, MA), where the number indicates the emission wavelength, as in our previous study.

### HER2 testing by IHC with QD‐conjugated antibody

The specimens were fixed in 10% formalin and embedded in paraffin, then cut into 3‐*μ*m thick sections and placed on glue‐coated glass slides. Sections were deparaffinized in xylene and hydrated with graded alcohols and distilled water. Antigen retrieval was performed using an autoclave (Tomy Sx‐500 High Pressure Steam Sterilizer; Tomy Seiko CO., LTD, Tokyo, Japan) in 10 mmol/L citrate buffer (pH, 6.0) and heated at 121°C for 5 min. Then samples were immunostained with 15 nmol/L QD‐conjugated trastuzumab or human IgG complexes for 3 h at 25°C. After being washed with phosphate‐buffered saline (PBS), the samples were incubated with DAPI for nuclear staining. The samples were washed with PBS and mounted with a mounting media (Aquatex; MERCK, Darmstadt, Germany). After that, the samples were observed with a single‐particle imaging system (Fig. S1). In addition, we performed the IHC with anti‐HER2 antibody which recognized the intracellular domain of HER2 protein (Fig. 1A) to investigate the difference between trastuzumab and the diagnostic anti‐HER2 antibody. The samples were immunostained with the anti‐HER2 antibody (Pathway, 4B5, Ventana) which recognized the intracellular domain of HER2 protein, the biotinylated secondary antibody, and streptavidin‐conjugated quantum dot 705 (Thermo Fisher Scientific, Waltham, MA) according to the manufacturer's instructions.

### Single‐particle imaging system

The optical system for observing the fluorescence of QDs consisted primarily of an epi‐fluorescent microscope (IX‐71; Olympus, Tokyo, Japan) with modifications, a Nipkow disk‐type confocal unit (CSU10; Yokogawa, Tokyo, Japan), and an electron multiplier type charge‐coupled device camera (EM‐CCD; Ixon DV887; Andor Technology, Belfast, UK) 27, 28. A PlanApo (X60, 1.40 NA; Olympus) objective lens was used for imaging. QDs were illuminated by a blue laser (488 nm wavelength, 50 mW, Spectra‐Physics). The laser‐excited fluorescence was filtered with a 695–740 nm band‐pass filter for imaging QDs and auto‐fluorescence of tissues or a 640–690 nm band‐pass filter for imaging auto‐fluorescence of tissues at identical focal plate and field. Images were obtained at exposure times of 200 msec or 20 sec.

### Data analysis

To quantitatively measure the particle numbers of QDs bound to cancer tissues, analysis was carried out as follows 15: The 512‐pixel‐square images, filtered with 695–740 nm band‐pass filter for QDs and auto‐fluorescence of tissues or a 640–690 nm band‐pass filter for the auto‐fluorescence, were taken at an exposure time of 20 sec. Each image was converted into a JPEG file. During the conversion, the autofluorescent signal of the image filtered with the 640–690 nm band‐pass filter (640–690 nm image) was adjusted to be about 1.2‐fold greater than that filtered with the 695–740 nm band‐pass filter (695–740 nm image). After file conversion, in order to visualize only the signal of the fluorescent QDs, the JPEG image of the 640–690 nm image was subtracted from that of 695–740 nm image using Adobe Photoshop image processing software. The fluorescent intensity of the QD signal in the subtracted image was analyzed as gray values using ImageJ software (http://rsb.info.nih.gov/ij/). The total fluorescent intensity in the image was defined as total‐QDs value. In the subtracted image, we also confirmed the fluorescent intensity in an area where there was an autofluorescent signal of zero. This indicates that, except for the signal of the QDs, there is no fluorescent signal in the background of the subtracted images. To precisely measure the number of the QD particles on the tissues, it is necessary to define the fluorescent intensity of a single QD. As QDs possessing the same fluorescent wavelength are uniform in size, the fluorescent intensity of QDs is proportional to the particle number. In addition, QD fluorescence is composed of fluorescent and nonfluorescent states called on‐ and off‐states. This fluorescent property results in blinking of QDs 29. When we measured the fluorescence of fresh QD particles after purchasing and analyzed their property, the results showed that the mean time of the off‐state during 20 sec of observation was about 4 sec and the calculated S.E.M. value was very low 30. If several QDs are aggregated, the mean time of the off‐state per unit time is shortened by aggregation of QDs because the on‐state and off‐state of each particle in the aggregate occurs randomly. Therefore, based on an off‐state time of 4 sec, we selected a single‐particle QD using each subtracted image and video image and measured the fluorescent intensity of the single QD particle (single‐QD value). In addition, the cell number in each image was measured using the DAPI image (300–600 cells were investigated in a patient sample). Then, the total‐QD value was divided by the single‐QD value to calculate the number of QD particles in a cell. Finally, we subtracted the value of the image labeled with QD‐conjugated human IgG (particle number/cell) from that labeled with QD‐conjugated trastuzumab, and obtained the precise mean particle number of QD‐conjugated trastuzumab bound specifically to a cancer cell.

### Statistical analysis

We randomly selected 37 patients with breast cancer, based on the result that 18 or more patient samples were required to statistically verify the strong correlation between IHC‐QD scores and FISH scores (*R* ≥ 7.0). The Pearson correlation method was used to compare the results between IHC‐QDs and FISH or TTP, considered as continuous variables. All statistical analyses were performed using the SAS software, JMP Pro 11. Two‐tailed *P* < 0.05 was considered statistically significant.

## Results

### IHC‐QDs using tissue samples with detailed clinical information

**Figure 2:**
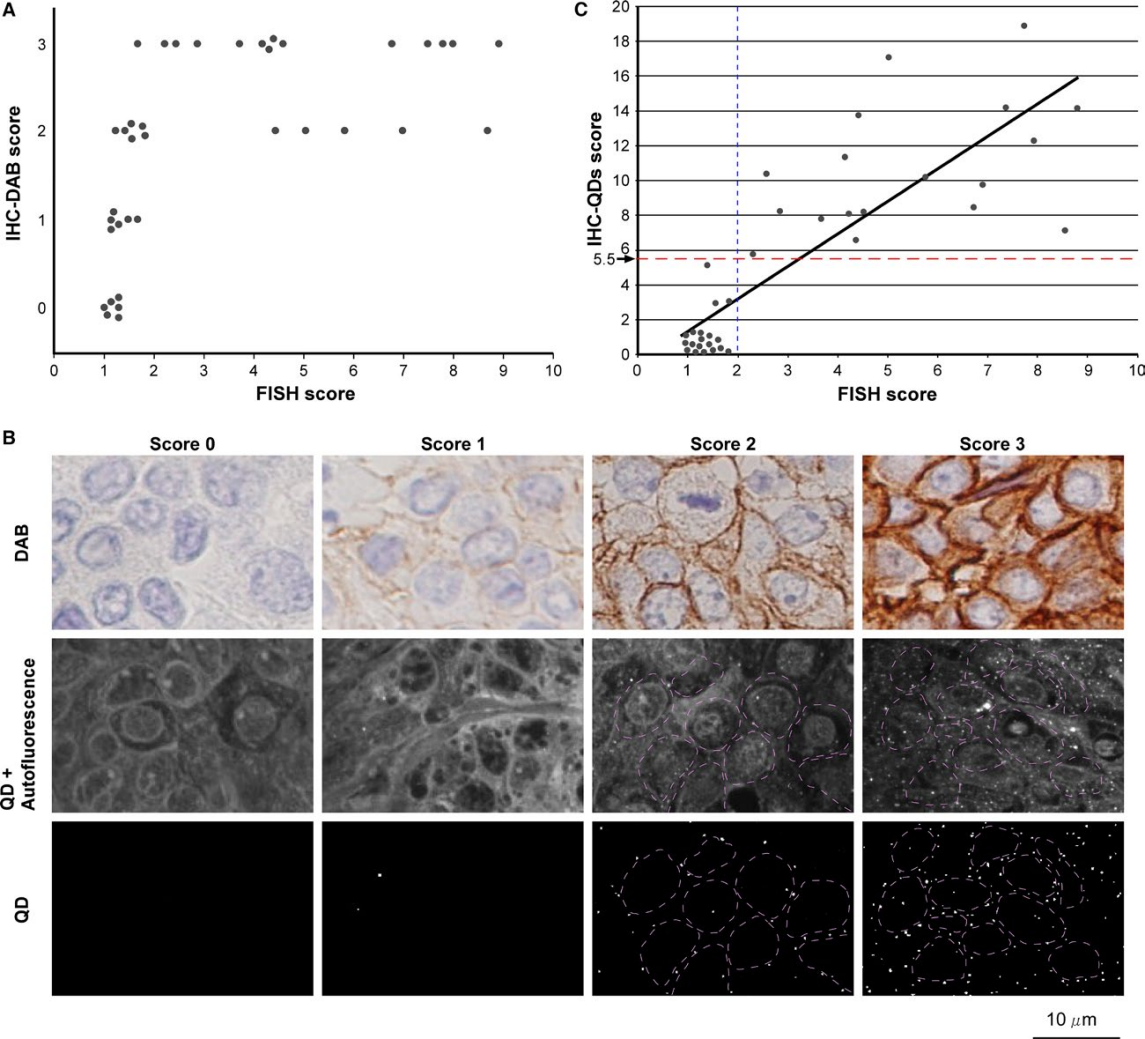
Development of IHC‐QDs against HER2. (A) Comparison between FISH and IHC‐DAB scores for 37 Cases with a score of 0 by IHC‐DAB ranged from 0.96 to 1.25 by FISH. Cases with a score of 1 had FISH scores from 1.12 to 1.64. Cases with a score of 2 varied widely, with FISH scores of 1.22–8.56, and those with a score of 3 also varied, from 1.65 to 8.76. The comparison of IHC‐DAB and FISH scores did not show a linear relationship, demonstrating that IHC‐DAB cannot precisely reveal the level of HER2 protein expression induced by its gene amplification. (B) Images of IHC stained with DAB‐labeled HER2 antibody (top row) or QD‐conjugated trastuzumab (center and bottom row) in samples with scores of 0, 1, 2, and 3 by IHC‐DAB. The center row shows the images observed with 695–740 nm band‐pass filter. The bottom row shows the images subtracting autofluorescence in tissues from the images of center row in order to visualize only QDs fluorescence (bright spots). Purple dotted lines show the outline of cancer cells. The microscope field of top and center row is different. (C) Comparison between FISH and IHC‐QDs scores for 37 cases. The IHC‐QDs score is the number of QD particles in a cell that reflects the level of HER2 protein expression in cancer cells. The straight‐line approximation curve shows that both quantitative scores are well correlated (*R* = 0.83). The blue dotted line shows the cut‐off line (2.0) of the FISH score. The score of 5.5 by IHC‐QDs represents the cut‐off level that we set in this study (red dotted line). DAB, 3,3′‐diaminobenzidine; HER2, human epidermal growth factor receptor 2; IHC, immunohistochemical.

To apply the IHC‐QDs to diagnosis of HER2‐positive breast cancer patients in the clinical setting, we selected tissue samples from 37 breast cancer patients for whom detailed clinical information was available (Table**** 1). The FISH and IHC‐DAB scores of these samples were compared. The result showed that both scores did not show a linear relationship (Fig. 2A), demonstrating that IHC‐DAB cannot represent the precise level of HER2 protein overexpression induced by its gene amplification.

**Table 1:** Characteristics of patients (*n* = 37)

Characteristics	
Age	
Median (range)	55 (25–88) year
Stage^a^ (%)	
I	13 (35)
II	13 (35)
III	9 (24)
IV	2 (6)
ER/PgR status (%)	
ER+ and/or PgR+	18 (49)
ER‐ and PgR‐	19 (51)
HER2 status by IHC‐DAB^a^ (%)	
Score 0	6 (16)
Score 1	6 (16)
Score 2	11 (30)
Score 3	14 (38)

a Stage grouping is based on TNM classification of malignant tumors seventh edition by the International Union Against Cancer (UICC) 42.

IHC‐DAB is conventional standard IHC by enzyme antibody technique using 3,3′‐Diaminobenzidine (DAB) for staining.

Figure 2B shows the IHC images immunostained with the DAB‐ or QDs‐labeling techniques. Samples with scores of 0, 1, 2, and 3 as assessed by IHC‐DAB are shown (Fig. 2B, upper panels). For IHC‐QDs, QDs images after autofluorescence‐subtraction showed only fluorescence of nanoparticles derived from the antigen‐antibody reaction (Fig. 2B, middle and lower panels). The QD particle number per cell in the image was evaluated by calculation using the value of single‐QD‐fluorescent intensity and cell number. The values for FISH, IHC‐QDs, and IHC‐DAB are listed in Table 2. We investigated the relationship between HER2 gene amplification and its protein expression by IHC‐QDs and the FISH score. Both scores were highly correlated in 37 tissue samples of breast cancers and are presented in a scatter plot diagram (*R* = 0.83, *P* < 0.001) (Fig. 2C). Eighteen of the 37 patient samples were characterized as HER2‐positive by FISH using the current cut‐off level of 2.0 (Fig. 2C, blue dotted line). In the HER2‐positive sample, all cases were shown to have an IHC‐QDs score higher than 5.7 (the average IHC‐QDs score for the 18 cases was 10.6). By contrast, in the other 19 HER2‐negative samples whose FISH scores were less than 2.0, most of IHC‐QDs scores were below 2.0 (the average IHC‐QDs score of these 19 patients was 0.82). If the cut‐off level of IHC‐QDs score was set at 5.5 in this study (Fig. 2C, red dotted line), the HER2‐positive or ‐negative diagnosis by IHC‐QDs corresponded exactly with that of the FISH score.

**Table 2:** Results of three diagnostic methods for HER2 expression, FISH, IHC‐DAB, and IHC‐QDs, for 37 cases

No.	Age	Stage	FISH	IHC‐DAB	IHC‐QDs
Case 1	80	I	0.96	0	0.62
Case 2	64	IIIB	1.40	2	5.27
Case 3	41	I	1.12	1	0.46
Case 4	55	IIA	7.70	3	18.81
Case 5	70	I	1.25	0	0.08
Case 6	74	I	4.38	2	13.71
Case 7	51	I	5.72	2	10.18
Case 8	41	IIIC	7.40	3	14.02
Case 9	54	IIIA	1.53	2	2.87
Case 10	42	IIB	1.77	2	0.13
Case 11	45	I	1.81	2	3.05
Case 12	68	IIIC	6.69	3	8.44
Case 13	80	I	1.52	2	0.06
Case 14	38	I	1.64	1	0.17
Case 15	55	IIIA	2.33	3	5.77
Case 16	25	IIIC	2.84	3	8.13
Case 17	44	IIIA	4.35	3	6.55
Case 18	79	IIB	1.14	1	0.54
Case 19	49	I	1.14	1	0.24
Case 20	65	IIA	1.25	0	0.25
Case 21	54	IIB	3.69	3	7.95
Case 22	88	I	1.16	0	0.20
Case 23	70	IIA	1.11	1	0.01
Case 24	64	I	1.25	0	0.67
Case 25	72	IIA	1.22	2	0.32
Case 26	53	I	1.02	0	0.34
Case 27	47	IIB	1.65	3	0.18
Case 28	63	IV	4.97	2	16.98
Case 29	54	IV	8.56	2	7.14
Case 30	38	IIB	8.76	3	14.04
Case 31	65	IIA	1.50	1	0.06
Case 32^a^	60	IIB	4.25	3	8.17
Case 33^a^	31	I	4.53	3	8.26
Case 34^a^	74	IIIC	6.90	2	9.69
Case 35^a^	32	IIB	2.55	3	10.26
Case 36^a^	73	IIIA	4.13	3	11.32
Case 37^a^	36	IIB	7.90	3	12.17

Stage grouping is based on TNM classification of malignant tumors seventh edition by the International Union Against Cancer (UICC) 42. IHC‐DAB is conventional standard IHC by enzyme antibody technique using 3,3′‐Diaminobenzidine (DAB) for staining.

a These cases were treated by trastuzumab therapy as a single agent for distant metastasis, and suitable for evaluation of trastuzumab efficacy using HER2 diagnostic methods.

### Development of IHC‐QDs against HER2

**Figure 3:**
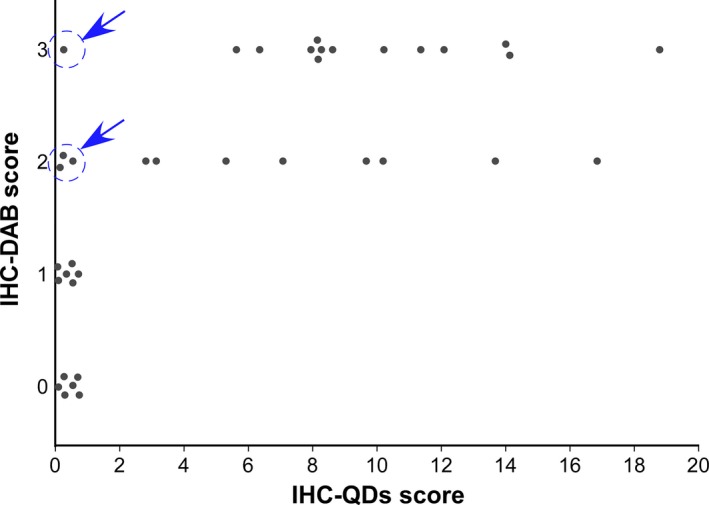
Comparison of IHC‐QDs and IHC‐DAB scores for 37 cases. Cases with a score of 0 by IHC‐DAB had scores of 0.08–0.67 with IHC‐QDs, those with a score of 1 had IHC‐QDs score from 0.01 to 0.54, those with a score of 2 varied widely, with IHC‐QDs scores of 0.06–16.98, and those with a score of 3 also varied, from 0.18 to 18.81. In some of the sample with IHC‐DAB score of 2 and 3, the binding of trastuzumab to these cancer cells is very weak (blue arrows) although they are candidate or trastuzumab therapy in present HER2 diagnostic criteria. Blue arrows show case 10, 13, 25, and 27. DAB, 3,3′‐diaminobenzidine; HER2, human epidermal growth factor receptor 2; IHC, immunohistochemical.

The accuracy of IHC‐QDs was further emphasized by comparing the results of two IHC methods (Fig. 3). Cases with an IHC‐DAB score of 0 had scores of 0.08–0.67 with IHC‐QDs and those with a score of 1 had IHC‐QDs scores of 0.01–0.54, which are both low values within a narrow range (Fig. 3 and Table 2). On the other hand, cases with a score of 2 had IHC‐QDs scores of 0.06–16.98, and cases with a score of 3 also varied from 0.18 to 18.81 (Fig. 3 and Table 2). These results comparing IHC‐QDs with IHC‐DAB showed that HER2 expression levels have a remarkably wide distribution in tumors that have scores of 2 and 3 by IHC‐DAB. Moreover, it is noteworthy that some of the samples that had scores of 2 and 3 by IHC‐DAB had an extremely low score using IHC‐QDs (Fig. 3, blue arrows), suggesting that the binding of trastuzumab to these samples is very weak in spite of the scores of 2 and 3. The gap between the IHC‐DAB and IHC‐QDs scores might be due to a difference in the epitopes of trastuzumab and antibodies for IHC‐DAB. Trastuzumab binds to the extracellular domain of HER2. On the other hand, the antibodies used for IHC‐DAB bind to its intracellular domain. It have been reported that various truncated forms of HER2 lack the extracellular domain. Additionally, it have shown that overexpression of MUC4 sealed the surface of the HER2 receptor. These effects on the extracellular domain of HER2 might inhibit the binding of trastuzumab to HER2 but not the binding of a diagnostic antibody and HER2. In this way, labeling trastuzumab directly for IHC is thought to be useful for selecting patients to treat with this drug (Fig. 1).

To investigate the difference between trastuzumab and the diagnostic anti‐HER2 antibody, we performed the IHC with anti‐HER2 antibody which recognizes the intracellular domain of HER2 protein (Fig. 1A and Fig. S2). In four cases (case 10, 13, 25, and 27) that had scores of 2 and 3 by IHC‐DAB had an extremely low score using IHC‐QDs, IHC with HER2‐intracellular domain‐recognizing anti‐HER2 antibody, biotinylated secondary antibody and streptavidin‐conjugated QDs (intIHC‐QDs) was done. The intIHC‐QDs scores of these cases varied from 16.85 to 164.19 (Table**** 3). As a result, case 13 and case 25 might have HER2 overexpressing tumors to which trastuzumab could not bind, but which were unfortunately diagnosed as HER2 positive. On the other hand, the scores of both IHC‐QDs and intIHC‐QDs were very low in case 10 and 27. For the experimental control, the samples of four cases (case 2, 4, 28, and 32) who had moderate or high scores of IHC‐QDs were stained by intIHC‐QDs (Table**** 3). The scores of case 13 (164.19) and case 25 (136.22) were similar to the score of case 2 (157.01) (Table**** 3), suggesting that effect on extracellular domain of HER2 in tissues of case 13 and 25 might inhibit the binding of trastuzumab to HER2 but not the binding of a HER2‐intracellular domain‐recognized antibody and HER2 (Fig. 1C).

**Table 3:** Comparison between IHC‐QDs and intIHC‐QDs in eight cases

No.	Age	Stage	FISH	IHC‐DAB	IHC‐QDs	intIHC‐QDs
Case 13	80	I	1.52	2	0.06	164.19
Case 10	42	IIB	1.77	2	0.13	16.85
Case 27	47	IIB	1.65	3	0.18	55.67
Case 25	72	IIA	1.22	2	0.32	136.22
Case 2	64	IIIB	1.40	2	5.27	157.01
Case 32	60	IIB	4.25	3	8.17	314.44
Case 28	63	IV	4.97	2	16.98	306.91
Case 4	55	IIA	7.70	3	18.81	234.26

Stage grouping is based on TNM classification of malignant tumors seventh edition by the International Union Against Cancer (UICC) 42. IHC‐DAB is conventional standard IHC by enzyme antibody technique using 3,3′‐Diaminobenzidine (DAB) for staining. intIHC‐QDs is IHC with QDs and anti‐HER2 antibody which recognize the intracellular domain of HER2 protein.

### Application of IHC‐QDs to diagnosis of HER2‐positive patients

**Figure 4:**
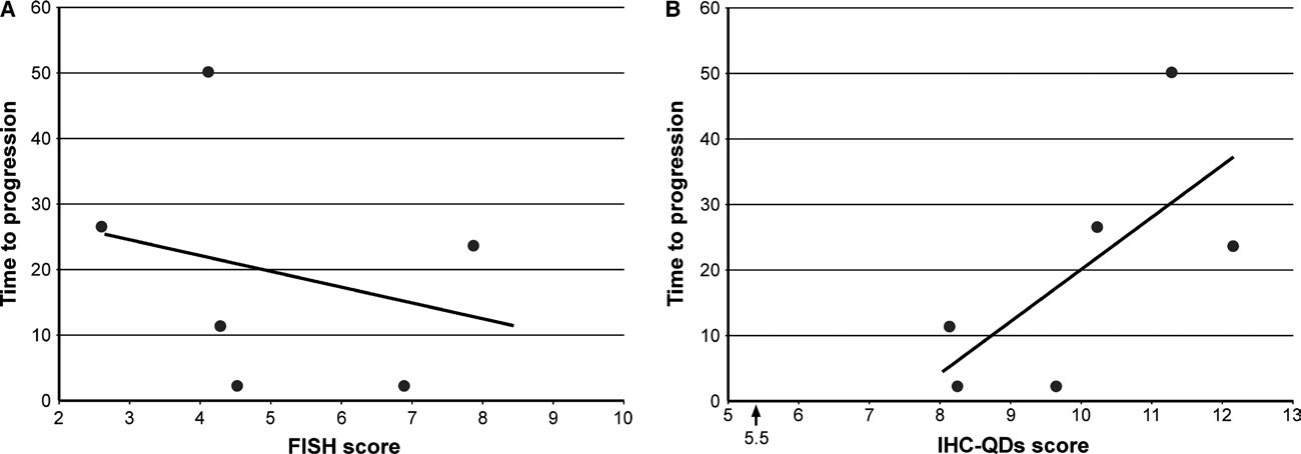
Comparison between IHC‐QDs or FISH and the therapeutic efficacy in six patients who were treated with trastuzumab therapy as a single agent for distant metastasis. (A) FISH score was not correlated with TTP, that is, time from the initiation of trastuzumab to disease progression. The response to trastuzumab could not be predicted by FISH score. (B) The good correlation between IHC‐QDs and TTP was observed in these cases (*R* = 0.69). This result suggests that the IHC‐QDs score can be a predictive factor for trastuzumab therapy. IHC, immunohistochemical; TTP, time to progression.

In this experiment, we detected six patients who were treated with trastuzumab therapy as a single agent for distant metastasis and examined the clinical efficacy of IHC‐QDs score as a predictive factor for trastuzumab therapy (Table**** 4). In these cases, the IHC‐QDs score was ranging from 8.17 to 12.17, and FISH score ranging from 4.13 to 7.90. To assess the usefulness of these scores as a predictive factor for trastuzumab therapy, we examined the relationship between IHC‐QDs or FISH score and time to progression (TTP) that is time from the initiation of trastuzumab to disease progression. IHC‐QDs score and TTP were well correlated in these six cases (*R* = 0.69, *P* < 0.01) (Fig. 4A). Conversely, the correlation between FISH score and TTP was not observed (Fig. 4B). These results suggest that response to trastuzumab therapy can be predicted by not FISH score but IHC‐QDs score.

**Table 4:** Correlation of IHC‐QDs or FISH with Response and TTP during trastuzumab therapy in six patients

No.	Age	Stage	ER/PgR	Metastatic site	FISH	IHC‐DAB	IHC‐QDs	Response^a^	TTP (month)
Case 32	60	IIB	−/−	Liver	4.25	3	8.17	SD	12
Case 33	31	I	−/−	Lung	4.53	3	8.26	PD	3
Case 34	74	IIIC	−/−	Liver	6.90	2	9.69	PD	3
Case 35	32	IIB	+/+	Lung	2.55	3	10.26	SD	27
Case 36	73	IIIA	−/−	Liver	4.13	3	11.32	PR	50
Case 37	36	IIB	−/−	Bone	7.90	3	12.17	SD	24

a Response criteria is based on RECIST guideline.

PR, partial response; SD, stable disease; PD, progression disease 24; TTP, Time to progression.

## Discussion

In this study, we developed an IHC method with trastuzumab conjugated with QDs, using tissue samples from patients. Labeling with fluorescent molecules is an effective way to use IHC as a quantitative method 31, 32 because the intensity of the fluorescent materials is proportional to the intensity of the photon excitation energy in an irreversible chemical reaction. However, general organic fluorescent molecules such as FITC, Alexa Fluors, and Cy‐5, have disadvantages arising from their poor photostability and tissue autofluorescence interference. QDs, which are bright and photostable nanoparticles, are a good tool for quantitative IHC. Several studies have succeeded in improving IHC with trastuzumab and other anti‐HER2 antibodies by conjugating with QDs 19, 20, 21, 22, 33, 34. In more recent studies, QDs conjugated to the single variable domain of anti‐HER2 antibodies or the novel near‐infrared QDs bioconjugated to anti‐HER2 antibodies were successfully used for immunolabeling breast cancer cells 19, 20. QDs developed using these methods might be potential biomarkers that are more sensitive than the conventional immunohistochemistry. However, previous studies did not use tissue samples from patients to obtain data pertaining to FISH score, the efficacy of trastuzumab, and clinical outcome. Therefore, an IHC method that bridges the gap between diagnostics and therapeutics has not yet been developed. Moreover, these previous IHC with QDs could not overcome the problem of tissue autofluorescence interference. The high intensity of tissue autofluorescence is comparable to the fluorescence intensity of QDs. Therefore, a quantitative analysis using only the fluorescence intensity of QDs in the presence of autofluorescence has been difficult to achieve.

Previous studies for IHC‐QDs against HER2 measured the amount of HER2 protein as the total fluorescent intensity of tissue filtered in a particular wavelength range. However, to estimate only the fluorescent signal of QDs in IHC‐QDs, it is more effective to describe the amount of fluorescence not as the total fluorescent intensity within a defined area of tissue but as QD‐particle number per cell. Because tissue samples have autofluorescence of various intensities, the total fluorescent intensity of the samples treated with IHC‐QDs will include both autofluorescence and the fluorescence of QDs, meaning that measurement of the total fluorescent intensity cannot exclude the possibility of a false‐positive signal. On the other hand, we recently developed a novel IHC‐QDs method using estimation of QD particle number by an image processing and single‐QD imaging 23. Our novel method consisted of subtracting autofluorescence of the tissue from total fluorescent intensity, including autofluorescence and QD fluorescence. The QD particle number in the subtracted image was then estimated by dividing the fluorescent intensity in the image by the intensity of single‐particle QDs (see Materials and Methods). The particle number of QDs is an absolute value and does not change depending on the individual optical system or in the presence of autofluorescence. Our user‐friendly IHC with trastuzumab conjugated with QDs was also developed using autofluorescence‐subtracted images and single‐particle QD imaging 23 (Fig. 1). In addition, some of the steps critical for IHC‐DAB, such as the blocking of endogenous peroxidase activity, secondary antibody reaction, and visualization of DAB, are not necessary for this procedure (Fig. 1). This is a powerful advantage of IHC‐QDs over IHC‐DAB because it enables a more rapid diagnosis. In the experiment, the omission of the blocking step was based on the results indicating that there was no difference in the IHC‐QD scores irrespective of whether the blocking step was performed. Instead of the blocking, we used control staining with QD‐conjugated human IgG to check the specificity of QD‐conjugated trastuzumab probes against HER2. For application of IHC‐QDs to other molecular marker, we previously targeted protease‐activated receptor 1 (PAR1) as a new biomarker of HER2‐negative patients. PAR1 is a G protein‐coupled receptor that plays an important role in metastatic processes in various cancers of the breast, colon, lung, pancreas, and prostate 35, 36. The immunostaining results for HER2‐negative human breast cancer tissue samples with anti‐PAR1 antibody‐conjugated QDs showed that the PAR1 expression level in cancer cells with a poor prognosis was strongly correlated with the prognosis of HER2‐negative breast cancer patients.

Many IHC studies have reported a correlation between HER2 gene amplification and its protein overexpression 37, 38. However, these results were based on qualitative measurement by IHC‐DAB 38 or a method used to investigate the level of HER2 protein in the serum without information about HER2 localization 38. Our results demonstrate that IHC‐QDs coupling with FISH is an outstanding method for precisely diagnosing HER2 status at both its gene and protein levels. To further apply the new method to increase the effectiveness of IHC‐QDs against HER2, we labeled trastuzumab with QDs rather than diagnostic anti‐human HER2 antibodies, owing to their different epitopes. We prepared monomerized trastuzumab‐conjugated QDs by mixing the trastuzumab and QDs in a molar ratio of approximately 3: 1. Then, the trastuzumab‐conjugated QDs were prepared using a Qdot 705 Antibody Conjugation Kit (Life Technologies) according to the manufacturer's instructions, as in a previous study 26. The diameters of the QD and monomer antibody are approximately 20 nm and 7–8 nm 39, respectively, yielding a volume ratio of approximately 20: 1. We previously estimated the number of monomer trastuzumab bound to the surface of a single QD by 0.8% agarose‐gel electrophoresis, and the sample of trastuzumab‐conjugated QDs was fractionated into three major bands 26. Approximately 60% of the trastuzumab‐conjugated QDs were conjugated with three monomer antibody fragments, 30% with two fragments, and 10% with a single fragment (mean value, 2.5 of monomer trastuzumab fragments per single QD) 31. These results demonstrate the following three features of trastuzumab‐conjugated QDs: (1) the monomer trastuzumab on trastuzumab‐conjugated QDs interacts with HER2 on the cell membrane in a one‐to‐one interaction; (2) the volume of monomer trastuzumab is considerably smaller than that of QD; and (3) the monomer trastuzumab binds to the QD surface at very low density. Therefore, these data strongly suggest that trastuzumab‐conjugated QDs interact with a single HER2 on the cell membrane.

Resistance to trastuzumab is becoming an increasingly important problem in clinical practice because the role of this molecular agent has been shown in recent studies not only for the management of metastatic breast cancers but also at the adjuvant setting for HER2‐overexpressing patients 3, 40, 41. In fact, about 70% of HER2‐positive patients receive unnecessary and inappropriate treatments as a result of resistance to trastuzumab therapy 3. Therefore, it is expected that pathological diagnosis by FISH and IHC can more accurately predict clinical response. The score of IHC‐QDs correlated proportionally with the FISH score and is a remarkable predictive factor for trastuzumab therapy in patients with breast cancer. We anticipate that our results will be confirmed by well‐controlled trials with larger sample sizes.

In summary, we performed the precisely quantitative IHC using trastuzumab‐conjugated QDs and single‐particle imaging analysis, and propose using IHC‐QDs score as a predictive factor for trastuzumab therapy. This new diagnostic method would be expected to contribute to the development of a therapeutic strategy and the realization of tailored treatment for breast cancer.

## Conflict of Interest

None declared.

## Supporting information


**Figure S1**. Images of IHC‐QDs stained with QD‐conjugated trastuzumab (A) or QD‐conjugated human IgG for control (B) in the same tumor.

**Figure S2.** Representative images for the comparison between IHC‐QDs and intIHC‐QDs in the same tumor. In this case (Case 13), the IHC‐QD score obtained for QD‐conjugated trastuzumab was extremely low (A). In contrast, the intIHC‐QD score obtained for HER2‐intracellular domain‐recognizing anti‐HER2 antibody, the biotinylated secondary antibody, and streptavidin‐conjugated QDs was high (B). Thus, this case might have had HER2‐overexpressing tumors to which trastuzumab could not bind, but which were unfortunately diagnosed as HER2‐positive. Purple dotted lines show the outline of cancer cells, as detected by a bright‐field image.
