# Supplementary figures and images for: Quantitative diagnosis of HER2 protein expressing breast cancer by single‐particle quantum dot imaging
Source: Cancer Med. 2016 Sep 26;5(10):2813–24. doi: 10.1002/cam4.898 (PMC5083734; doi:10.1002/cam4.898)

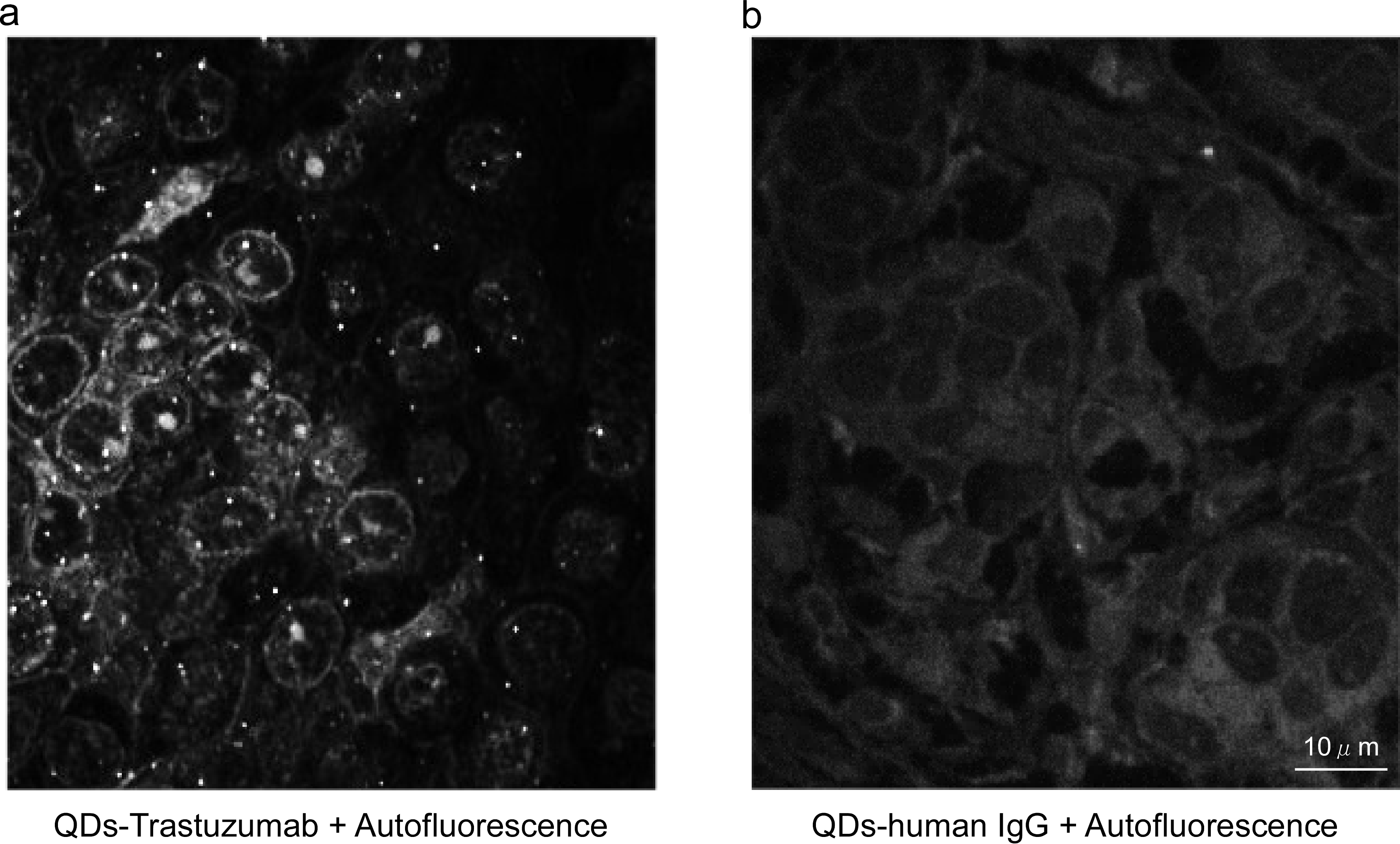

Supplement: Supplementary file 1 — Figure S1. Images of IHC‐QDs stained with QD‐conjugated trastuzumab (A) or QD‐conjugated human IgG for control (B) in the same tumor. [file CAM4-5-2813-s001.tif]

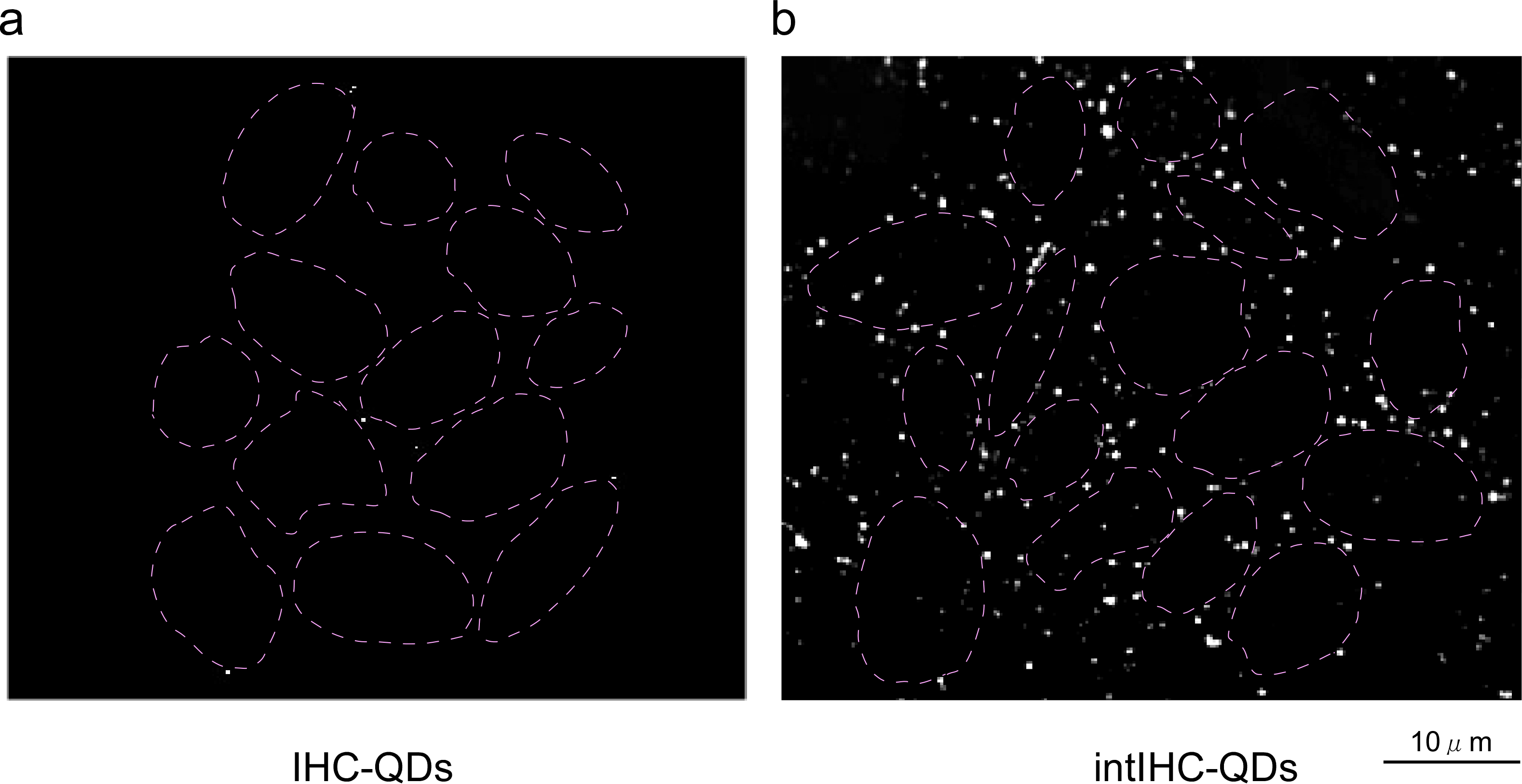

Supplement: Supplementary file 2 — Figure S2. Representative images for the comparison between IHC‐QDs and intIHC‐QDs in the same tumor. In this case (Case 13), the IHC‐QD score obtained for QD‐conjugated trastuzumab was extremely low (A). In contrast, the intIHC‐QD score obtained for HER2‐intracellular domain‐recognizing anti‐HER2 antibody, the biotinylated secondary antibody, and streptavidin‐conjugated QDs was high (B). Thus, this case might have had HER2‐overexpressing tumors to which trastuzumab could not bind, but which were unfortunately diagnosed as HER2‐positive. Purple dotted lines show the outline of cancer cells, as detected by a bright‐field image. [file CAM4-5-2813-s002.tif]
